# NEDD9 Facilitates Hypoxia-Induced Gastric Cancer Cell Migration via MICAL1 Related Rac1 Activation

**DOI:** 10.3389/fphar.2019.00291

**Published:** 2019-04-04

**Authors:** Shuo Zhao, Pengxiang Min, Lei Liu, Lin Zhang, Yujie Zhang, Yueyuan Wang, Xuyang Zhao, Yadong Ma, Hui Xie, Chenchen Zhu, Haonan Jiang, Jun Du, Luo Gu

**Affiliations:** ^1^Department of Physiology, Nanjing Medical University, Nanjing, China; ^2^Jiangsu Key Lab of Cancer Biomarkers, Prevention and Treatment, Collaborative Innovation Center For Cancer Personalized Medicine, Nanjing Medical University, Nanjing, China; ^3^Department of Biochemistry and Molecular Biology, Nanjing Medical University, Nanjing, China; ^4^Department of Implantology, Changzhou Stomatological Hospital, Changzhou, China; ^5^School of Basic Medical Science, Nanjing Medical University, Nanjing, China

**Keywords:** MICAL1, NEDD9, migration, gastric cancer, Rac1

## Abstract

**Aims and Hypothesis:** NEDD9 is highly expressed in gastric cancer and has a significant involvement in its pathogenesis. However, the mechanism behind hypoxia-promoted cancer cell migration and its regulation because of NEDD9 is still unknown. The aim of this study is to investigate the involvement of NEDD9 in gastric cancer cell migration under hypoxia and explore the underlying potential molecular mechanisms.

**Methods:**

Cell motility was measured by wound healing and transwell assay. NEDD9 and MICAL1 expressions were examined by western blot analysis. Interaction between NEDD9 and MICAL1 was assessed by immunohistochemistry and co-immunoprecipitation assay, respectively. Cells were transfected with plasmids or siRNA to upregulate or downregulate the expression of NEDD9 and MICAL1. Rac1, Cdc42, and RhoA activation was assessed by pulldown assay.

**Results:**

The mRNA and protein level of NEDD9 increased as a result of hypoxia in gastric cancer cell lines BGC-823 and SGC-7901 while decreased levels of NEDD9 caused reduced cell migratory potential in response to hypoxia. Hypoxia also caused the enhancement of MICAL1 expression. Furthermore, it was revealed that there is a positive correlation between NEDD9 and MICAL1 protein while hypoxia played role in increasing their interaction. Under hypoxic conditions, silencing of NEDD9 caused reduction in the stability of MICAL1 protein, while depletion of MICAL1 also inhibited the migration of NEDD9-overexpressing gastric cancer cells. In addition, silencing of NEDD9 or MICAL1 expression reversed the increased GTP forms of Rac1 and Cdc42 in hypoxic cells. However, only the upregulation of Rac1-GTP level was observed in gastric cancer cells that were already overexpressed by MICAL1.

**Conclusion:**

In all, it is concluded that MICAL1 is regulated by NEDD9 that facilitates hypoxia-induced gastric cancer cell migration via Rac1-dependent manner.

## Introduction

Neural precursor cell expressed developmentally downregulated protein 9 (NEDD9) also known as HEF1, Cas-L, CASS2 is a member of the Crk-associated substrate (CAS) protein family. NEDD9 was first described to be expressed in the brain of the mouse in embryonic stage—where its expression was seen developmentally downregulated (Kumar et al., 1992). As a non-catalytic scaffolding protein containing multiple docking sites, NEDD9 is involved in connecting external stimulus signals with downstream signaling molecules. Therefore, it plays a critical role in assembling signaling cascades and regulating multiple cellular processes including tumor progression (Nikonova et al., 2014; Shagisultanova et al., 2015).

It has been documented that NEDD9 is strongly associated with development of multiple types of cancer (Jin et al., 2014; Wang et al., 2014; Zhou et al., 2017). NEDD9 is overexpressed in gastric cancer tissues (Liu et al., 2014). The upregulation of NEDD9 can lead to malignant transformation of normal human gastric epithelial cells (Feng et al., 2015). Growing evidence suggests that overexpression of NEDD9 can increase the risk of metastasis of cancer cells. For example, NEDD9 is highly enriched in focal adhesion and interacts with FAK and Src, which can initiate cell migration and invasion (Sima et al., 2013; Bradbury et al., 2014). Upregulation of NEDD9 is reported to activate Rac1 GTPase and drive mesenchymal-mode movement of cancer cells (Sanz-Moreno et al., 2008). In addition, NEDD9 is positively correlated with the expression of mesenchymal-type marker proteins such as vimentin and Zeb, while E-cadherin is opposite (Feng et al., 2015). NEDD9 is also involved in canonical Wnt/β-catenin pathway-mediated colonic cell migration and cancer progression (Li et al., 2011). Understanding the NEDD9 regulatory pathway in cell migration will provide not only new insights into cancer metastasis but also therapeutic targets for gastric cancer. However, the mechanism underlying the effect of NEDD9 on gastric cancer cell motility has not been fully investigated.

Hypoxia is a powerful selective driver of aggressive tumor behavior (Bertout et al., 2008). One of the key regulators of cellular response to hypoxia is the increased expression level of hypoxia-inducible factor 1α (HIF-1α). Stable HIF1α expression can modulate cellular metabolism and increase angiogenesis (Hockel and Vaupel, 2001). NEDD9 has been identified as a novel HIF-1α-regulated gene which can mediate hypoxia-induced colorectal cancer cell migration (Kim et al., 2010). In addition, NEDD9 was also found selectively induced in neurons after transient global ischemia (Sasaki et al., 2005). However, involvement of NEDD9 with gastric cancer cell motility and relative mechanisms under hypoxic condition is still unknown. Molecules interacting with Cas L (MICALs) are redox enzymes that are crucial for cytoskeleton dynamics (Vanoni, 2017; Yoon and Terman, 2018). Our previous work identified a novel link between MICAL1 and RAB upon EGF stimulation and found that MICAL1 was essential for maintaining invasive phenotype of breast cancer cells (Deng et al., 2016b). Unfortunately, the regulator(s) of MICAL1 in gastric cancer is still unclear. NEDD9 was identified to interact with MICAL1 by far western screening analysis (Suzuki et al., 2002) providing a basis for further exploring the role of MICAL1 in NEDD9-induced alteration of cancer cell function.

The present study is a continuation of our previous study where we have proven that MICAL1 exerts a significant growth and invasion-promoting effects on breast cancer cells (Deng et al., 2016b, 2018). The results here demonstrated that hypoxia promoted gastric cancer cell migration by positively regulating the expression of NEDD9 and its subsequent binding to MICAL1. In addition, NEDD9/MICAL1 also promotes cell migration in Rac1-dependent manner. These findings revealed a novel relationship between NEDD9 and MICAL1 in the context of hypoxia-induced gastric cancer cell migration.

## Materials and Methods

### Ethics Statement

All immunohistochemistry assays with human tumor specimens were conducted under the institutional guidelines of Jiangsu Province.

### Cell Culture

Human gastric cancer cell lines (BGC-823, SGC-7901) and HEK-293T cells were obtained from the Cell Biology Institute of Chinese Academy of Sciences (Shanghai, China). Cells were cultured in Dulbecco’s modified Eagle’s medium (DMEM, high glucose) (Hyclone, Thermo Scientific, Waltham, MA, United States) supplemented with 10% (v/v) fetal bovine serum (FBS) (Gibco, Carlsbad, CA, United States) and antibiotics (100 U/mL streptomycin and 100 μg/mL penicillin) (Invitrogen, Carlsbad, CA, United States) in a humidified incubator at 37°C with 5% CO_2_. Cells were grown on coverslips for fluorescence staining and on plastic dishes for protein extraction.

For hypoxia, cells were exposed to a continuous flow of a humidified mixture of 1% O2, 5% CO2, and 94% N2 at 37°C for the indicated time.

### Plasmids and siRNAs

Human full-length NEDD9 cDNA was amplified from pBluescriptR-NEDD9 plasmid (Youbio, Hunan, China) using the following primer set, sense: 5′-AAGGGTACCGAGCTGGATCCATGAAGTATAAGAATCTTATGGCA-3′ and antisense: 5′-TGCTGGATATCTGCAGAATTCTCAGAACGTTGCCATCTC-3′. In these primers, BamHI and EcoRI restriction site sequences have been underlined. The full-length MICAL1 DNA was amplified from pOTB7-MICAL1 plasmid using the following primer set, sense: 5′-CCCAAGCTTGCCACCATGGCTTCACCTACCTCCA-3′, antisence: 5′-CCAACTCGAGGCCCTGGGCCCCTGTCCCCAAGGCCA-3′. In these primers, HindIII and XhoI restriction site sequences have been underlined. The PCR products were cloned into the pCMV-C-HA vector (Clontech, Palo Alto, CA, United States). All constructions were ensured by sequencing. The cells were grown in six-well plates until approximately 80% confluence, and then transiently transfected with those plasmids by using FuGENE HD Transfection Reagent (Promega, Madison, WI, United States) according to the manufacturer’s instructions.

The siRNAs were synthesized and purified by GenePharma (Shanghai, China), and the siRNAs specifically targeting NEDD9 were as follows: #1, 5′-GAGGCGUUCAGUUUCUUGAdTdT-3′, #2, 5′-CCAAGAACAAGAGGUAUAUd TdT-3′, and #3, 5′-GAUGGGAUCAACCGAUUGUdTdT-3′. siRNAs specifically targeting MICAL1 were as follows: 5′-CUCGGUGCUAAGAAGUUCUdTdT-3′. Cells were transfected with siRNA using Lipofectamine 2000 (Thermo Fisher Scientific, Waltham, MA, United States) according to the transfection method provided by the manufacturer. After transfection with plasmid or siRNA for 36 h, the cells were cultured in hypoxia, and then treated with cycloheximide (CHX) (Sigma, St. Louis, MO, United States) at the indicated time points.

### Cell Wound Healing and Transwell Assay

After transfection with indicated exogenous materials when the cells reached approximately 95–100% confluent, a scratch was made manually in the monolayer of cells by using a 10 μL pipette tip. The cells were washed with PBS and then incubated in fresh medium with or without hypoxia. The wounded cellular monolayer was permitted to heal for 12 h. Photographs of wound healing were taken using microscope (Carl Zeiss Meditec, Jena, Germany).

Transwell assay were performed using a 24-well cell culture insert with 8 μm pores (Millipore, Billerica, MA, United States). Cells were harvested, washed, and suspended in DMEM without FBS, then seeded on the upper chamber with density of 4 × 10^4^/200 μL. Cells were allowed to get attached to the membrane for about 30 min. The lower chamber was filled with 600 μL DMEM with 10% FBS. After incubation for 12 h, the cells were fixed, and stained with 0.1% crystal violet for 5 min. Then the cells on the upper surface of the membrane were removed and the number of stained cells on the lower surface of the membrane was counted in photos taken under an inverted microscope (TS100; Nikon, Tokyo, Japan).

### Co-immunoprecipitation Assay

Co-immunoprecipitation assay were performed as previously described (Deng et al., 2016a). Briefly, cell lysates were incubated with antibody at 4°C for 4 h. Antibody-bound complexes were precipitated with protein A+G agarose beads (Beyotime, Nantong, China) and rinsed with PBS. Then agarose-associated protein complexes were dissolved in SDS loading buffer and analyzed by immunoblotting analysis.

### Immunoblotting Analysis

Protein extraction from the sample and concentration determination of whole cells was performed as previously described (Duan et al., 2016). Briefly, the cells were lyzed in RIPA buffer containing phenylmethanesulfonyl fluoride (PMSF) and protease inhibitor cocktail. Equal amounts of proteins were resolved on SDS polyacrylamide gels and transferred to nitrocellulose membrane. The resulting blots were blocked with 5% non-fat dry milk and probed with antibodies. The following antibodies were used: GAPDH (KangChen, Shanghai, China), β-actin (Santa Cruz, Santa Cruz, CA, United States), MICAL1 (proteintech, Hubei, China), NEDD9 (Santa Cruz), FLAG (Abways, Shanghai, China), Rac1 (BD, Franklin Lakes, NJ, United States), RhoA, Cdc42, and HA antibodies (Cell Signaling, Danvers, MA, United States). Protein bands were detected by incubating with HRP-conjugated antibodies (Santa Cruz) and developed using ECL reagent (Millipore). Digital images of the positive bands were obtained and analyzed with Quantity One (Bio-Rad, Hercules, CA, United States).

### Pulldown Assay

Rac1, Cdc42, and RhoA activity was measured by pulldown assay (Chen et al., 2018). Active RhoA was pulldown by GST-RBD beads and active Cdc42/Rac1 was pulldown by PAK-CRIB beads. In brief, protein lysates were centrifuged, supernatant was collected in new tubes containing beads precoupled with GST–PBD or PAK-CRIB, and incubated under rotation at 4°C for 30 min. Then, the beads were washed and the proteins bound on the beads were separated by SDS-PAGE. The amounts of active RhoA, Cdc42, and Rac1 were determined by immunoblotting analysis.

### Measurement of ROS

2′,7′-Dichlorofluorescein diacetate (CM-H2DCFDA) (Invitrogen, Carlsbad, CA, United States), a ROS-specific fluorescent probe, was used to determine the intracellular ROS levels. After exposing it to hypoxia for 4 h, the cells were stained with 5 μM CM-H2DCFDA for 15 min at 37°C. After washing with PBS, the cover slips were mounted on glass slides. Images were collected using an Olympus BX51 microscope coupled with an Olympus DP70 digital camera.

### Immunohistochemistry

Tumor specimens were obtained from Outdo biotech (Shanghai, China). Thirty primary human gastric tumor samples and their corresponding paracancerous tissue samples were used for immunohistological staining in our study. The sections were deparaffinized and rehydrated. Peroxidase blocking was done with 3% H_2_O_2_ in methanol for 15 min at 37°C. Antigen retrieval was performed by transferring the sections into EDTA buffer (pH 8.0). The sections were then blocked by goat serum and applied with MICAL1 (proteintech, Hubei, China) or NEDD9 (absin, Shanghai, China) antibody at 4°C overnight. Next, the sections were treated with the secondary antibody (MXB, Fujian, China) for 1 h at room temperature. After counterstaining with hematoxylin, the slides were mounted and photographs were obtained using an Olympus BX51 microscope. Reagents for immunohistochemistry were all obtained from ZSGB-BIO (Beijing, China). The Immuno Reactive Score (IRS) was calculated as intensity of the staining reaction multiplied by the percentage of the number of positive cells as previously described(Dumitru et al., 2013; Medale-Giamarchi et al., 2013).

### CCK8 Assay

Cell proliferation was determined by using cell counting kit-8 kits (bimake, Houston, TX, United States) according to the manufacturer’s instructions. Cells were seeded at a density of 5 × 10^3^ cells per well into 96-well plate (Corning), 10 μL of the CCK-8 solution was added to each well of the plate. After 1 h, the absorbance was conducted at 450 nm using a microplate absorbance reader (Bio-Tek, Elx800, United States).

### RT-qPCR

Total RNAs were prepared using TRIzol reagent (Invitrogen). cDNA was synthesized using equal amounts of RNA (0.5 μg) from each sample. qPCR was performed on the ABI StepOne^TM^ Real-Time PCR System (Applied Biosystems, Foster City, CA, United States) using GoTaq qPCR Master Mix assay (Promega). The gene expression levels were calculated with Rt (2^-ΔΔCT^) values by StepOne Software v 2.1 (Applied Biosystems). The following primers were used to amplify: β-actin: 5′-CATGTACGTTGCTATCCAGGC-3′ (sense) and 5′-CTCCTTAATGTCACGCACGAT-3′ (antisense); MICAL1:5′GGCACTCGGTGCTAAGAAGTT-3′ (sense) and 5′-CCCCAGTGAATTTCCACCCC-3′ (antisense); NEDD9: 5′-GACCGTCATAGAGCAGAACAC-3′ (sense) and 5′-TGCATGGGACCAATCAGAAGC-3′ (antisense).

### Statistical Analysis

All experiments were repeated at least three times and whole data are presented as mean ± SD. The significance of difference in two groups was analyzed by Student’s *t*-test. *P* < 0.05 represents statistical significance and *P* < 0.01 represents sufficiently statistical significance (two-tailed). Pearson correlation test was used indicate the association between MICAL1 and NEDD9 protein expressions in immunohistochemistry analysis.

## Results

### Hypoxia Promotes NEDD9 Protein Accumulation in Gastric Cancer Cells

**FIGURE 1:**
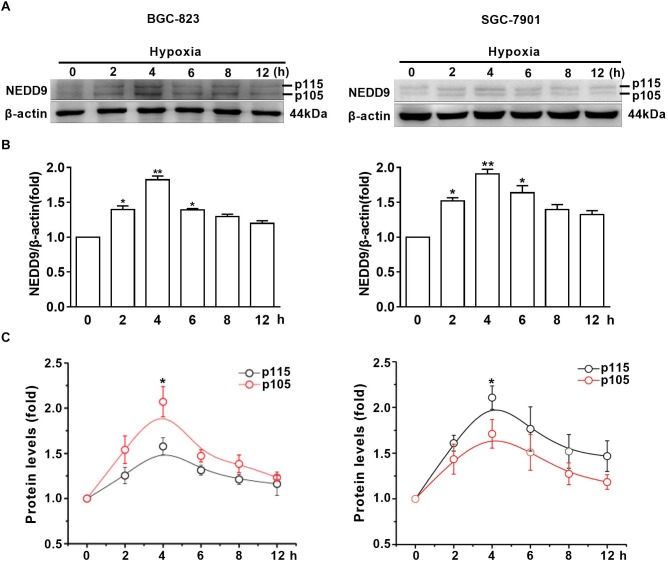
Hypoxia increases NEDD9 protein expression. **(A)** Gastric cancer cell lines BGC-823 and SGC-7901 were exposed to hypoxia for 12h and NEDD9 protein levels were determined by immunoblotting analysis. **(B)** NEDD9 was quantified and normalized against β-actin. ^∗^*P* < 0.05, ^∗∗^*P* < 0.01, referring to the difference between cells treated with and without hypoxia. **(C)** Quantification of p105 and p115 isoforms of NEDD9 during hypoxia incubation.^∗^*P* < 0.05, referring to the difference between p105 and p115 isoforms during hypoxia.

To assess the effect of hypoxia on NEDD9 expression in gastric cancer cells, SGC-7901 and BGC-823 cells were cultured under hypoxia for the indicated time. As it has been previously shown in other cell types, NEDD9 protein usually appears as 105 and 115 kD isoforms (Latasa et al., 2016). The results in Figure 1A show that hypoxia induced an increase in both NEDD9 isoforms in gastric cancer cells within 2 h and peaked at 4 h of hypoxia, then returned to the basal level at 12 h. The whole western blot picture of NEDD9 is available in Supplementary Figure S1. The elevated levels of NEDD9 mRNA were detected by qPCR (Supplementary Figure S2). Although hypoxia increased both bands in each doublet, modified their proportion and changed the ratio between them. In BGC-823 cells, p115 isoform accounted for about 50% of total NEDD9 in resting cells. While hypoxia incubation caused lowering of p115 isoform proportion as compared to control group, therefore the proportion of p105 isoform became higher in BGC-823. In contrast, more proportion of p115 isoform produced in SGC-7901 cells under hypoxic condition (Figure 1B,C). These results confirmed that hypoxia is an inducer of NEDD9 expression in gastric cancer cells.

### Hypoxia Stimulates Gastric Cancer Cell Migration Through NEDD9

**FIGURE 2:**
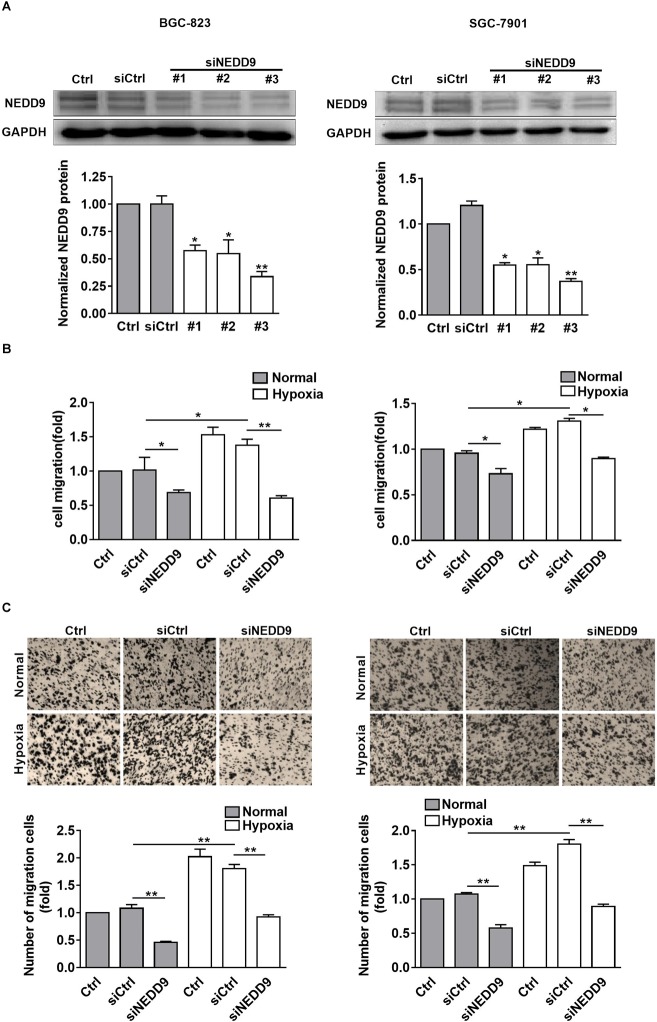
Effect of NEDD9 on hypoxia-induced gastric cancer cell migration. **(A)** BGC-823 cells (left) and SGC-7901 cells (right) were transfected with control siRNA or NEDD9 siRNA. After 48 h, immunoblotting analysis was performed to detect the expression of NEDD9 and GAPDH. The bands were quantified and normalized against GAPDH. ^∗^*P* < 0.05, ^∗∗^*P* < 0.01, referring to the difference between cells transfected with control siRNA or NEDD9 siRNA. The migratory capacity of those cells transfected with NEDD9 siRNA under hypoxia were evaluated by wound healing assay **(B)** and transwell assay **(C)**. ^∗^*P* < 0.05, ^∗∗^*P* < 0.01.

As shown in Figure 2A, knockdown of NEDD9 with siRNA reduced the NEDD9 expression in both SGC-7901 and BGC-823 cells, indicating the efficiency of the siRNA targeting NEDD9 in the present study. We noticed that siRNA #3 was most effective among the three NEDD9 siRNAs; therefore, it was selected for the following study. To further examine whether hypoxia stimulates gastric cancer cell migration in NEDD9-dependent manner, we investigated SGC-7901 and BGC-823 cell migration using wound closure assay after transfecting these cells with NEDD9 siRNA. Under hypoxia, the cell migration rate got increased significantly as compared to the cells under normal condition. However, in NEDD9-silenced cells, such stimulatory effect of hypoxia on cell migration was greatly inhibited (Figure 2B). Cell migration was also assessed by transwell migration assay. The migrated cell number was increased two folds in hypoxia treated cultures as compared to control group of cells. Knockdown of NEDD9 significantly decreased the cell migration rate in both normoxia- or hypoxia-treated gastric cancer cells (Figure 2C). NEDD9 knockdown had no significant effect on proliferative capacity in gastric cancer cells under hypoxia for 12 h, evidenced by the CCK8 assay (Supplementary Figure S3), ruling out the effect of proliferation on cell migration in the indicated time period. In all, the results above indicated that the increased expression of NEDD9 was essential for hypoxia-stimulated gastric cancer cell migration.

### MICAL1 Is Required for Hypoxia-Induced Cell Migration

**FIGURE 3:**
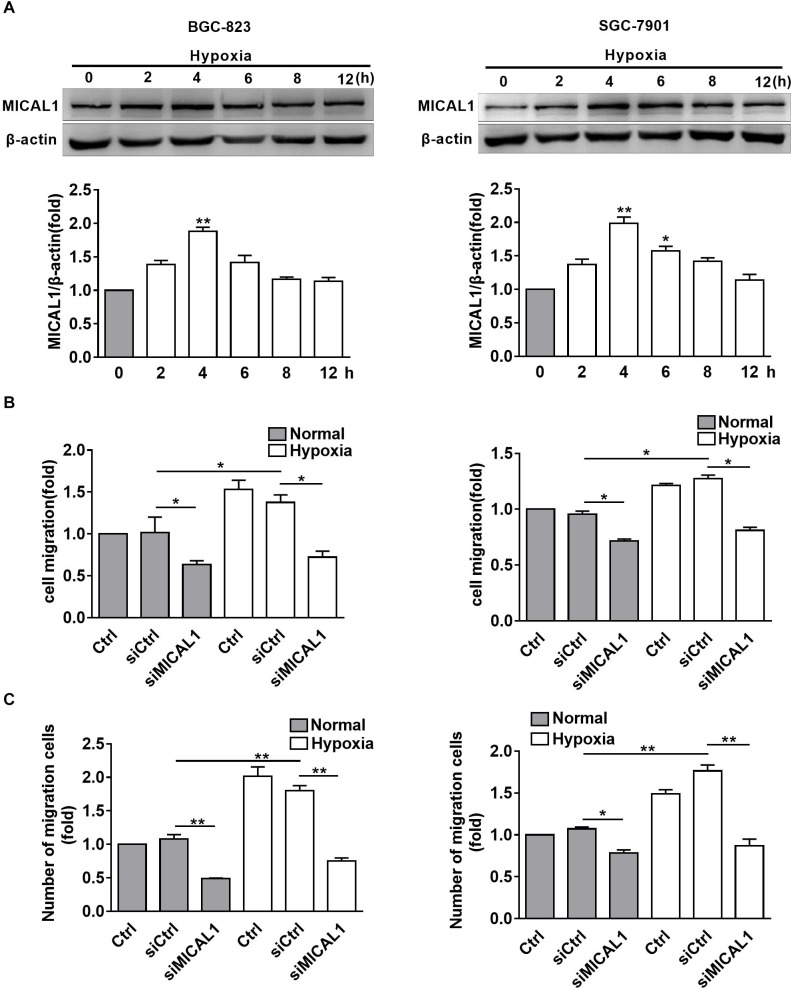
MICAL1 is essential for hypoxia-induced cell migration. **(A)** BGC-823 cells (left) and SGC-7901 cells (right) were treated with hypoxia for 12 h and the expression of MICAL1 protein levels was determined by immunoblotting analysis. The bands were quantified and normalized against β-actin. ^∗^*P <* 0.05, ^∗∗^*P <* 0.01 referring to the difference between cells treated with and without hypoxia. The migratory capacity of those cells transfected with MICAL1 siRNA under hypoxia was evaluated by wound healing assay **(B)** and transwell assay **(C)**. ^∗^*P* < 0.05, ^∗∗^*P* < 0.01.

It has been found that EGF-induced breast cancer cell invasion is MICAL1-dependent, which forms complexes with RAB35 (Deng et al., 2016b). Based on the finding that NEDD9 interacts with MICAL1 by far western screening (Suzuki et al., 2002), we further investigated whether MICAL1 was also involved in hypoxia-stimulated gastric cancer cell migration. The levels of MICAL1 mRNA were not changed significantly under hypoxia by qPCR analysis (Supplementary Figure S2). Immunoblotting analysis showed that, in both gastric cancer cells, the amount of MICAL1 was increased significantly after hypoxia incubation with maximal activation at 4 h, and it declined toward basal levels after 8 h (Figure 3A). Furthermore, knockdown of MICAL1 repressed the hypoxia-promoted gastric cancer cell migratory phenotype (Figure 3B,C).

### NEDD9 Prevents MICAL1 Degradation

**FIGURE 4:**
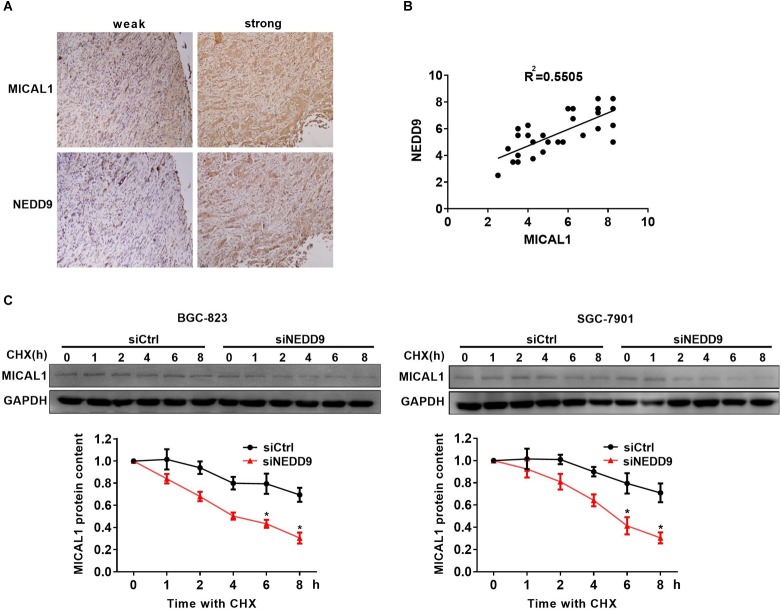
NEDD9 binds to MICAL1 and prevents its degradation. **(A)** Using serial sections of the same sample, representative gastric cancer tissue stained for MICAL1 and NEDD9 (weak and strong staining) are shown. **(B)** The scatterplot of correlated protein levels between MICAL1 and NEDD9 were shown (*n* = 30). **(C)** After blocking protein synthesis with CHX (10 μg/mL) for the indicated times, the hypoxic cells transfected with control siRNA or NEDD9 siRNA were lyzed and MICAL1 levels were determined by immunoblotting analysis. GAPDH was used as control. ^∗^*P* < 0.05 referring to the difference between cells treated with control siRNA or NEDD9 siRNA.

Immunohistochemistry analysis in the gastric cancer tissue microarray showed a positive correlation between NEDD9 and MICAL1 expressions (*r* = 0.55) (Figure 4A,B). The result suggests a possibility that the upregulation of NEDD9 may contribute to the MICAL1 expression in gastric cancer cells. It is reported that NEDD9 overexpression correlates with poor prognosis of gastric cancer (Liu et al., 2014; Zhang et al., 2015), However, in our study, due to the limited number of samples, we were unable to demonstrate the significant interaction between NEDD9, MICAL1, and gastric cancer progression (data not shown). MICAL1 is a redox enzyme that is crucial for ROS generation. Knockdown of either NEDD9 or MICAL1 in gastric cancer cells inhibited hypoxia-induced ROS production (Supplementary Figure S4). Furthermore, the co-immunoprecipitation study on both Flag-NEDD9 and HA-MICAL1 overexpressed in HEK293 cells indicated the direct binding of MICAL1 and NEDD9 (Supplementary Figure S5A). The interaction between MICAL1 and NEDD9 was also confirmed by co-immunoprecipitation assay, which showed the endogenous MICAL1 interacted with NEDD9 in SGC-7901 and BGC-823 cells. Moreover, under hypoxic conditions, the interaction increased in those gastric cancer cells (Supplementary Figure S5B). To determine whether NEDD9 could regulate stability of MICAL1, we blocked the protein synthesis by CHX. As shown in Figure 4C, under hypoxia, the expression levels of MICAL1 were dramatically decreased over the course of 8 h by depleted NEDD9 expression in comparison with control cells. In all, these data suggested that by interaction with the MICAL1, NEDD9 maintained MICAL1 stability under hypoxia by reducing its degradation in gastric cancer cells.

### Silencing of NEDD9/MICAL1 Blocks Hypoxia-Induced Rho GTPases Activation

**FIGURE 5:**
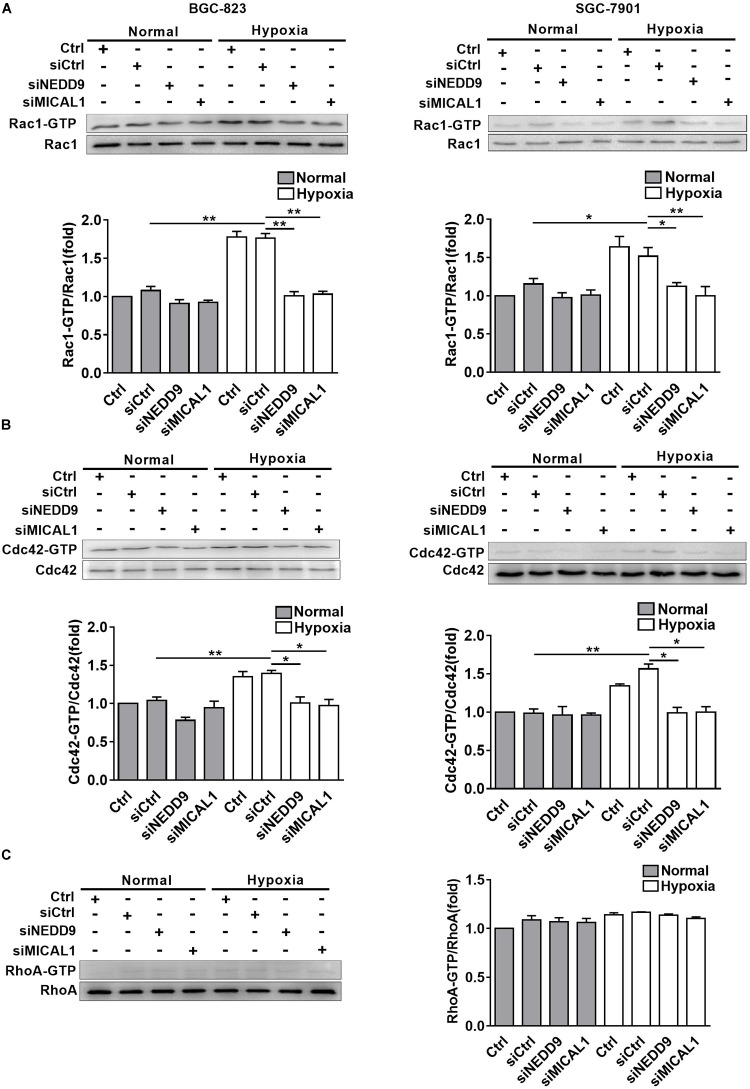
Effect of NEDD9 and MICAL1 on hypoxia-induced Rho-GTPases activation. **(A–C)** BGC-823 and SGC-7901 cells were transfected with MICAL1 siRNA or NEDD9 siRNA, and then those cells were exposed to hypoxia for 4 h. Protein extraction from cells was analyzed by immunoblotting analysis. The GTP-bound form of endogenous Rac1 **(A)**, Cdc42 **(B)**, and RhoA **(C)** were precipitated and examined by immunoblotting analysis. All the experiments were repeated thrice. ^∗^*P* < 0.05, ^∗∗^*P* < 0.01.

The members of Rho GTPases, Rac1, Cdc42, and RhoA, have been considered as classical cytoskeleton regulators associated with cancer cell migratory phenotype. To verify that NEDD9 and MICAL1 could impact hypoxia-mediated activity of those Rho GTPases, we transfected cells with NEDD9 siRNA or MICAL1 siRNA, and then examined their effects on Rac1, Cdc42, and RhoA. The efficiency of the siRNA targeting NEDD9 or MICAL1 is presented in Supplementary Figure S6. Results from pulldown assay showed that hypoxia incubation increased both Rac1 and Cdc42 activation in SGC-7901 and BGC-823 cells. Furthermore, increased activation of Rac1 and Cdc42 under hypoxia was attenuated by either NEDD9 or MICAL1 depletion (Figure 5A,B). Since RhoA activation in BGC-823 cells was not significantly affected by hypoxia as well as NEDD9 or MICAL1 depletion, we did not test it again in SGC-7901 cells (Figure 5C). Collectively, these data indicated that NEDD9 and MICAL1 contribute to hypoxia-induced Rac1 and Cdc42 activation.

### Rac1 Mediates NEDD9/MICAL1 Induced Gastric Cancer Migration

**FIGURE 6:**
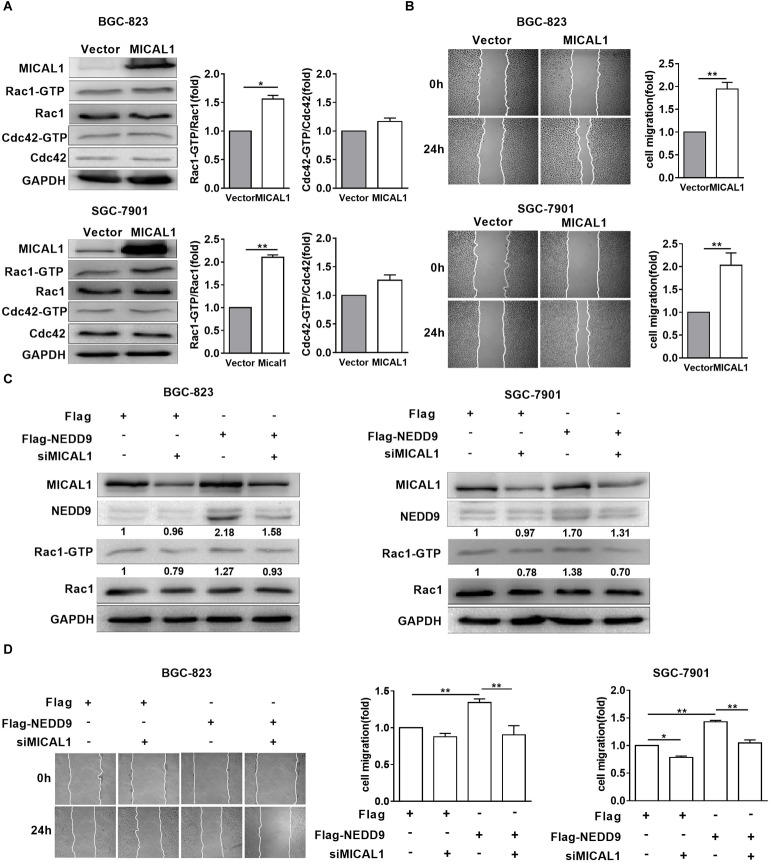
Rac1 mediates MICAL1/NEDD9 induced gastric cancer migration. **(A)** BGC-823 and SGC-7901 cells were transfected with MICAL1 plasmids where total cellular proteins were extracted and analyzed for GTP-bound form of Rac1 and Cdc42. ^∗^*P* < 0.05, ^∗∗^*P* < 0.01. **(B)** Cells were transfected with MICAL1 plasmids or empty vectors, and then wound healing assay were performed to evaluate the migration of both cells. ^∗∗^*P* < 0.01. **(C)** BGC-823 and SGC-7901 cells were transfected with NEDD9 plasmids and/or MICAL1 siRNA. After 48 h, extracted protein from cells was analyzed for NEDD9 and Rac1-GTP expressions. **(D)** Results of wound healing assay showed that knockdown of MICAL1 delayed cell migration in BGC-823 and SGC-7901 cells which overexpressed NEDD9. ^∗^*P* < 0.05, ^∗∗^*P* < 0.01.

MICAL1 plasmid significantly increased the MICAL1 expression in gastric cancer cells, confirming the efficiency of the MICAL1 overexpression protocol in the present study (Figure 6A). It is noted that MICAL1 overexpression only increased Rac1 activation rather than Cdc42 activation (Figure 6A), suggesting that the role of MICAL1 in mediating cell migration may depend on Rac1 activation. The role of MICAL1 in regulating cell migration was further confirmed by the facts that MICAL1 overexpression promoted gastric cell migration rate (Figure 6B). As shown in Figure 6C, Rac1 activation was increased in NEDD9-overexpressed gastric cancer cells. Rac1-GTP levels were increased by 1.27-folds in BGC-823 cells and by 1.38-folds in SGC-7901 cells However, knockdown of MICAL1 attenuated NEDD9-induced Rac1 activation, Rac1-GTP levels were decreased to 0.93-folds in BGC-823 cells and 0.70-folds in SGC-7901 cells when compared with control group. Knockdown of MICAL1 also prevented NEDD9-stimulated cell migration in both BGC-823 and SGC-7901 cells (Figure 6D). Collectively, these results revealed that NEDD9/MICAL1 may act on Rac1 to affect hypoxia-induced gastric cancer cell migration.

## Discussion

Globally, gastric cancer is the fifth most-common cancer and the third leading cause of death (McGuire, 2016). The prognosis of gastric cancer is poor, due to the fact that the tumor has already metastasized by the time of diagnosis. Emerging evidence indicated that hypoxia is one of the major factors for gastric cancer progression (Fujikuni et al., 2014). However, the molecular mechanisms underlying metastasis in gastric cancer cell under hypoxia were not fully understood. It is well accepted that NEDD9 expression is sensitive to multiple stimuli such as TGF-beta (Morimoto et al., 2014), retinoic acid (Latasa et al., 2016), estrogen (Bradshaw et al., 2011), and progesterone receptor overexpression (Richer et al., 2002). NEDD9 mRNA was also confirmed to be upregulated by hypoxia in stem cells derived from umbilical cord blood and bone marrow (Martin-Rendon et al., 2007). NEDD9 was overexpressed in gastric cancer patients (Karabulut et al., 2015; Zhang et al., 2015). The present study provides evidence that hypoxia promotes NEDD9 protein expression in gastric cancer cells. Meanwhile, silencing of NEDD9 suppressed the increased cell migration stimulated by hypoxia, confirming that NEDD9 was involved in the migration of gastric cancer cells under hypoxia. Moreover, we noticed here that NEDD9 appeared as two main protein phospho-forms of 105 and 115 kDa. Both phospho-forms were identified to be highly expressed by retinoic acid stimulation (Latasa et al., 2016); 105 kDa NEDD9 is already tyrosine phosphorylated and undergoes further phosphorylation modifications to produce a 115 kDa hyper-phosphorylated protein that is tyrosine and serine phosphorylated. The increase in the proportion of the 105 kDa isoform in BGC-823 and 115 kDa isoform in SGC-7901 indicated a relative different increment of NEDD9 phosphorylation status in various types of gastric cancer cells under hypoxia.

By far western screening analysis, MICAL interacts with SH3 domain of NEDD9 through its proline-rich PPKPP sequence (Suzuki et al., 2002). However, least data are available about the relationship between NEDD9 and MICAL1 in gastric cancer cells. MICAL1, a multidomain flavoenzyme, is strongly involved in the mechanisms that promote breast cancer cell proliferation and invasion (Deng et al., 2016b, 2018). By interacting with cytoskeletal components, MICAL1 has also been proven to be involved in the terminal steps of cytokinesis (Fremont et al., 2017). Recent study highlighted that MICAL1 deficiency in oral squamous cell carcinoma arrested MMP9 secretion, vimentin, and E-cadherin levels, consistent with increased N-cadherin (Grauzam et al., 2018). It suggests the possibility that MICAL1 participated in the control of gastric cancer cell migration. By analysis of gastric cancer specimens, we noticed a positive correlation between NEDD9 and MICAL1 protein expressions. So it is interesting to investigate the role of NEDD9 and MICAL1 in hypoxia-treated gastric cancer cells. *In vitro*, hypoxia induced MICAL1 protein level in a time-dependent fashion in gastric cancer cells. Silencing of NEDD9 significantly accelerated MICAL1 degradation and reduced cell migration rate under hypoxia. In contrast, MICAL1 deletion inhibited NEDD9-mediated migration in gastric cancer cell lines. Therefore, it was identified that hypoxia-induced NEDD9 expression has the ability to maintain MICAL1 protein level by reducing its degradation, thereby maintaining gastric cancer cell migratory properties. In the present work, we observed that NEDD9 bound directly to MICAL1 in gastric cancer cells and this interaction increased under hypoxic condition. The results suggested that NEDD9 might enhance its binding to MICAL1 and therefore decrease its degradation under hypoxia. The precise mechanisms by which NEDD9 regulates MICAL1 levels still needed to be identified.

Next we examined the potential effectors under NEDD9/MICAL1 for cell migration under hypoxia. Cdc42, Rac1, and RhoA, members of Rho GTPase family, are the most common regulators of actin cytoskeleton dynamics and cell movement. Similar to others findings (Xue et al., 2006; Du et al., 2011), here we found that hypoxia elevated both Rac1-GTP and Cdc42-GTP levels, but not RhoA-GTP in gastric cancer cells. Furthermore, knockdown of NEDD9 or MICAL1 prevented hypoxia-induced upregulation of Rac1 and Cdc42 activity and cell migration. Interestingly, only Rac1 activity was increased in MICAL1-overexpressed gastric cancer cells. Rac1 cycles between active GTP-bound and inactive GDP-bound states. In its active state, it promotes cell migration by driving cell polarization and lamellipodia formation (Raftopoulou and Hall, 2004; Parri and Chiarugi, 2010). NEDD9 plays an essential role in mesenchymal-mode cell migration via Rac1 activation (Jones et al., 2017), and MICAL has the ability to induce redox changes in Rho GTPase family (Ventura and Pelicci, 2002). It may be reasonable to speculate that NEDD9/MICAL1-enhanced hypoxic gastric cancer cell motility is mediated at least in part through Rac1 activation.

It remains unclear how MICAL1 causes the activation of Rac1 upon hypoxia. Hypoxia is able to upregulate PI3K/Akt signaling activation in breast cancer cells (Du et al., 2011). We previously demonstrated that MICAL1 enhanced PI3K/Akt activation (Deng et al., 2016b), and PI3K/Akt has been identified as a upstream activator of mTOR, p70S6K1, as well as GSK-3β. All of the above are linked with Rac1 activation (Aslan et al., 2011; Rom et al., 2012; Hall et al., 2018). As we know that the structure of MICAL1 contains monooxygenase domain, therefore, it participates in controlling intracellular ROS generation. Many studies suggested that ROS-mediated cancer cell migration is associated with PI3K/Akt activation (Yang et al., 2011; Huang et al., 2013). Therefore, it is understood that MICAL1 might stimulate Rac1 activation via PI3K/Akt signaling in gastric cancer cells. In all, we identified a novel link between NEDD9 and MICAL1 in accelerating gastric cancer cell motility under hypoxia. Hypoxia increased NEDD9 expression and its interaction with MICAL1, thereby preventing MICAL1 degradation. Furthermore, we observed that hypoxia stimulated Rac1 activation, which may serve as an important mediator for NEDD9/MICAL1-facilitated gastric cancer cell migration. The function and regulation of NEDD9/MICAL1 expression may be of major clinical importance and may provide new insights for the discovery of novel therapeutic targets.

## Author Contributions

JD designed the study. SZ, PM, LL, LZ, YZ, YW, XZ, YM, and HJ performed the experiments. SZ, HX, and CZ performed the statistical analysis. JD and LG drafted the manuscript and supervised the experimental work. All authors read and approved the final manuscript.

## Conflict of Interest Statement

The authors declare that the research was conducted in the absence of any commercial or financial relationships that could be construed as a potential conflict of interest.

## Supplementary Material

FIGURE S1 Raw data of NEDD9 expression under hypoxia. The membranes were exposed for protein bands. NEDD9 expression was detected three times **(A–C)**.

FIGURE S2 Effect of hypoxia on **(A)** NEDD9 and **(B)** MICAL1 mRNA expressions. Gastric cancer cells BGC-823 and SGC-7901 were exposed to hypoxia for 12 h and NEDD9 and MICAL1 mRNA levels were determined by qPCR. Afterward, NEDD9 and MICAL1 mRNAs were quantified and normalized against β-actin. ^∗^*P* < 0.05, ^∗∗^*P* < 0.01, referring to the difference between cells treated with and without hypoxia.

FIGURE S3 Effect of NEDD9 and MICAL1 on cell proliferation under hypoxia. **(A)** BGC-823 and **(B)** SGC-7901 cells were transfected with MICAL1 siRNA and NEDD9 siRNA and then they were exposed to hypoxia for 12 h. The cells were incubated with CCK8 and analyzed for cell proliferation.

FIGURE S4 Effect of NEDD9 and MICAL1 on ROS production under hypoxia. **(A)** BGC-823 and **(B)** SGC-7901 cells were transfected with MICAL1 siRNA or NEDD9 siRNA and then exposed to hypoxia for 4 h. Afterward, the cells were incubated with CM-H2DCFDA and analyzed for ROS generation. ^∗^*P* < 0.05, ^∗∗^*P* < 0.01.

FIGURE S5 NEDD9 interacts with MICAL1 in gastric cancer cells. **(A)** Immunoprecipitation assay was performed in HEK293T cells that co-transfected with HA-tagged MICAL1 or/and FLAG-tagged NEDD9. **(B)** Co-immunoprecipitation assay was performed which shows binding of endogenous NEDD9 to MICAL1 in BGC-823 and SGC-7901 cells under hypoxia.

FIGURE S6 Knockout efficiency of NEDD9 and MICAL1. **(A)** BGC-823 and **(B)** SGC-7901 cells were transfected with MICAL1 siRNA or NEDD9 siRNA, and then exposed to hypoxia for 4 h. Protein extracted from cells was analyzed by immunoblotting analysis.

## References

[B1] Aslan J. E. Tormoen G. W. Loren C. P. Pang J. McCarty O. J. (2011). S6K1 and mTOR regulate Rac1-driven platelet activation and aggregation. *Blood* 118 3129–3136. 10.1182/blood-2011-02-331579 21757621 PMC3175787

[B2] Bertout J. A. Patel S. A. Simon M. C. (2008). The impact of O2 availability on human cancer. *Nat. Rev. Cancer* 8 967–975. 10.1038/nrc2540 18987634 PMC3140692

[B3] Bradbury P. Bach C. T. Paul A. O’Neill G. M. (2014). Src kinase determines the dynamic exchange of the docking protein NEDD9 (neural precursor cell expressed developmentally down-regulated gene 9) at focal adhesions. *J. Biol. Chem.* 289 24792–24800. 10.1074/jbc.M113.544106 25059660 PMC4155648

[B4] Bradshaw L. N. Zhong J. Bradbury P. Mahmassani M. Smith J. L. Ammit A. J. (2011). Estradiol stabilizes the 105-kDa phospho-form of the adhesion docking protein NEDD9 and suppresses NEDD9-dependent cell spreading in breast cancer cells. *Biochim. Biophys. Acta* 1813 340–345. 10.1016/j.bbamcr.2010.11.018 21145356

[B5] Chen Y. Zhang L. Liu L. Sun S. Zhao X. Wang Y. (2018). Rasip1 is a RUNX1 target gene and promotes migration of NSCLC cells. *Cancer Manag. Res.* 10 4537–4552. 10.2147/CMAR.S168438 30349386 PMC6190810

[B6] Deng W. Gu L. Li X. Zheng J. Zhang Y. Duan B. (2016a). CD24 associates with EGFR and supports EGF/EGFR signaling via RhoA in gastric cancer cells. *J. Transl. Med.* 14:32. 10.1186/s12967-016-0787-y 26830684 PMC5439121

[B7] Deng W. Wang Y. Gu L. Duan B. Cui J. Zhang Y. (2016b). MICAL1 controls cell invasive phenotype via regulating oxidative stress in breast cancer cells. *BMC Cancer* 16:489. 10.1186/s12885-016-2553-1 27430308 PMC4950114

[B8] Deng W. Wang Y. Zhao S. Zhang Y. Chen Y. Zhao X. (2018). MICAL1 facilitates breast cancer cell proliferation via ROS-sensitive ERK/cyclin D pathway. *J. Cell. Mol. Med.* 22 3108–3118. 10.1111/jcmm.13588 29524295 PMC5980113

[B9] Du J. Xu R. Hu Z. Tian Y. Zhu Y. Gu L. (2011). PI3K and ERK-induced Rac1 activation mediates hypoxia-induced HIF-1alpha expression in MCF-7 breast cancer cells. *PLoS One* 6:e25213. 10.1371/journal.pone.0025213 21980400 PMC3181265

[B10] Duan B. Cui J. Sun S. Zheng J. Zhang Y. Ye B. (2016). EGF-stimulated activation of Rab35 regulates RUSC2-GIT2 complex formation to stabilize GIT2 during directional lung cancer cell migration. *Cancer Lett.* 379 70–83. 10.1016/j.canlet.2016.05.027 27238570

[B11] Dumitru C. A. Bankfalvi A. Gu X. Zeidler R. Brandau S. Lang S. (2013). AHNAK and inflammatory markers predict poor survival in laryngeal carcinoma. *PLoS One* 8:e56420. 10.1371/journal.pone.0056420 23409183 PMC3567070

[B12] Feng J. Zhao J. Xie H. Yin Y. Luo G. Zhang J. (2015). Involvement of NEDD9 in the invasion and migration of gastric cancer. *Tumour Biol.* 36 3621–3628. 10.1007/s13277-014-2999-1 25577245

[B13] Fremont S. Hammich H. Bai J. Wioland H. Klinkert K. Rocancourt M. (2017). Oxidation of F-actin controls the terminal steps of cytokinesis. *Nat. Commun.* 8:14528. 10.1038/ncomms14528 28230050 PMC5331220

[B14] Fujikuni N. Yamamoto H. Tanabe K. Naito Y. Sakamoto N. Tanaka Y. (2014). Hypoxia-mediated CD24 expression is correlated with gastric cancer aggressiveness by promoting cell migration and invasion. *Cancer Sci.* 105 1411–1420. 10.1111/cas.12522 25174257 PMC4462374

[B15] Grauzam S. Brock A. M. Holmes C. O. Tiedeken J. A. Boniface S. G. Pierson B. N. (2018). NEDD9 stimulated MMP9 secretion is required for invadopodia formation in oral squamous cell carcinoma. *Oncotarget* 9 25503–25516. 10.18632/oncotarget.25347 29876004 PMC5986644

[B16] Hall G. Lane B. M. Khan K. Pediaditakis I. Xiao J. Wu G. (2018). The human FSGS-causing ANLN R431C mutation induces dysregulated PI3K/AKT/mTOR/Rac1 signaling in podocytes. *J. Am. Soc. Nephrol.* 29 2110–2122. 10.1681/ASN.2017121338 30002222 PMC6065096

[B17] Hockel M. Vaupel P. (2001). Tumor hypoxia: definitions and current clinical, biologic, and molecular aspects. *J. Natl. Cancer Inst.* 93 266–276. 10.1093/jnci/93.4.266 11181773

[B18] Huang J. S. Cho C. Y. Hong C. C. Yan M. D. Hsieh M. C. Lay J. D. (2013). Oxidative stress enhances Axl-mediated cell migration through an Akt1/Rac1-dependent mechanism. *Free Radic. Biol. Med.* 65 1246–1256. 10.1016/j.freeradbiomed.2013.09.011 24064382

[B19] Jin Y. Li F. Zheng C. Wang Y. Fang Z. Guo C. (2014). NEDD9 promotes lung cancer metastasis through epithelial-mesenchymal transition. *Int. J. Cancer* 134 2294–2304. 10.1002/ijc.28568 24174333

[B20] Jones B. C. Kelley L. C. Loskutov Y. V. Marinak K. M. Kozyreva V. K. Smolkin M. B. (2017). Dual targeting of mesenchymal and amoeboid motility hinders metastatic behavior. *Mol. Cancer Res.* 15 670–682. 10.1158/1541-7786.MCR-16-0411 28235899 PMC5457705

[B21] Karabulut M. Alis H. Afsar C. U. Karabulut S. Kocatas A. Oguz H. (2015). Serum neural precursor cell-expressed, developmentally down regulated 9 (NEDD9) level may have a prognostic role in patients with gastric cancer. *Biomed. Pharmacother.* 73 140–146. 10.1016/j.biopha.2015.06.010 26211595

[B22] Kim S. H. Xia D. Kim S. W. Holla V. Menter D. G. Dubois R. N. (2010). Human enhancer of filamentation 1 is a mediator of hypoxia-inducible factor-1alpha-mediated migration in colorectal carcinoma cells. *Cancer Res.* 70 4054–4063. 10.1158/0008-5472.CAN-09-2110 20442290 PMC2871069

[B23] Kumar S. Tomooka Y. Noda M. (1992). Identification of a set of genes with developmentally down-regulated expression in the mouse brain. *Biochem. Biophys. Res. Commun.* 185 1155–1161. 10.1016/0006-291X(92)91747-E 1378265

[B24] Latasa M. J. Jimenez-Lara A. M. Cosgaya J. M. (2016). Retinoic acid regulates Schwann cell migration via NEDD9 induction by transcriptional and post-translational mechanisms. *Biochim. Biophys. Acta* 1863(7 Pt A), 1510–1518. 10.1016/j.bbamcr.2016.04.009 27085739

[B25] Li Y. Bavarva J. H. Wang Z. Guo J. Qian C. Thibodeau S. N. (2011). HEF1, a novel target of Wnt signaling, promotes colonic cell migration and cancer progression. *Oncogene* 30 2633–2643. 10.1038/onc.2010.632 21317929 PMC3164309

[B26] Liu Y. Wang D. Zhao K. L. Zhu J. W. Yin H. B. Wei Y. Z. (2014). NEDD9 overexpression correlates with poor prognosis in gastric cancer. *Tumour Biol.* 35 6351–6356. 10.1007/s13277-014-1839-7 24664584

[B27] Martin-Rendon E. Hale S. J. Ryan D. Baban D. Forde S. P. Roubelakis M. (2007). Transcriptional profiling of human cord blood CD133+ and cultured bone marrow mesenchymal stem cells in response to hypoxia. *Stem Cells* 25 1003–1012. 10.1634/stemcells.2006-0398 17185612

[B28] McGuire S. (2016). World cancer report 2014. Geneva, Switzerland: World Health Organization, International Agency for Research on Cancer, WHO Press, 2015. *Adv. Nutr.* 7 418–419. 10.3945/an.116.012211 26980827 PMC4785485

[B29] Medale-Giamarchi C. Lajoie-Mazenc I. Malissein E. Meunier E. Couderc B. Berge Y. (2013). RhoB modifies estrogen responses in breast cancer cells by influencing expression of the estrogen receptor. *Breast Cancer Res.* 15:R6. 10.1186/bcr3377 23339407 PMC3672819

[B30] Morimoto K. Tanaka T. Nitta Y. Ohnishi K. Kawashima H. Nakatani T. (2014). NEDD9 crucially regulates TGF-beta-triggered epithelial-mesenchymal transition and cell invasion in prostate cancer cells: involvement in cancer progressiveness. *Prostate* 74 901–910. 10.1002/pros.22809 24728978

[B31] Nikonova A. S. Gaponova A. V. Kudinov A. E. Golemis E. A. (2014). CAS proteins in health and disease: an update. *IUBMB Life* 66 387–395. 10.1002/iub.1282 24962474 PMC4111207

[B32] Parri M. Chiarugi P. (2010). Rac and Rho GTPases in cancer cell motility control. *Cell Commun. Signal.* 8:23. 10.1186/1478-811X-8-23 20822528 PMC2941746

[B33] Raftopoulou M. Hall A. (2004). Cell migration: Rho GTPases lead the way. *Dev. Biol.* 265 23–32. 10.1016/j.ydbio.2003.06.003 14697350

[B34] Richer J. K. Jacobsen B. M. Manning N. G. Abel M. G. Wolf D. M. Horwitz K. B. (2002). Differential gene regulation by the two progesterone receptor isoforms in human breast cancer cells. *J. Biol. Chem.* 277 5209–5218. 10.1074/jbc.M110090200 11717311

[B35] Rom S. Fan S. Reichenbach N. Dykstra H. Ramirez S. H. Persidsky Y. (2012). Glycogen synthase kinase 3beta inhibition prevents monocyte migration across brain endothelial cells via Rac1-GTPase suppression and down-regulation of active integrin conformation. *Am. J. Pathol.* 181 1414–1425. 10.1016/j.ajpath.2012.06.018 22863953 PMC3463628

[B36] Sanz-Moreno V. Gadea G. Ahn J. Paterson H. Marra P. Pinner S. (2008). Rac activation and inactivation control plasticity of tumor cell movement. *Cell* 135 510–523. 10.1016/j.cell.2008.09.043 18984162

[B37] Sasaki T. Iwata S. Okano H. J. Urasaki Y. Hamada J. Tanaka H. (2005). Nedd9 protein, a Cas-L homologue, is upregulated after transient global ischemia in rats: possible involvement of Nedd9 in the differentiation of neurons after ischemia. *Stroke* 36 2457–2462. 10.1161/01.STR.0000185672.10390.30 16210561

[B38] Shagisultanova E. Gaponova A. V. Gabbasov R. Nicolas E. Golemis E. A. (2015). Preclinical and clinical studies of the NEDD9 scaffold protein in cancer and other diseases. *Gene* 567 1–11. 10.1016/j.gene.2015.04.086 25967390 PMC4458429

[B39] Sima N. Cheng X. Ye F. Ma D. Xie X. Lu W. (2013). The overexpression of scaffolding protein NEDD9 promotes migration and invasion in cervical cancer via tyrosine phosphorylated FAK and SRC. *PLoS One* 8:e74594. 10.1371/journal.pone.0074594 24058594 PMC3776827

[B40] Suzuki T. Nakamoto T. Ogawa S. Seo S. Matsumura T. Tachibana K. (2002). MICAL, a novel CasL interacting molecule, associates with vimentin. *J. Biol. Chem.* 277 14933–14941. 10.1074/jbc.M111842200 11827972

[B41] Vanoni M. A. (2017). Structure-function studies of MICAL, the unusual multidomain flavoenzyme involved in actin cytoskeleton dynamics. *Arch. Biochem. Biophys.* 632 118–141. 10.1016/j.abb.2017.06.004 28602956

[B42] Ventura A. Pelicci P. G. (2002). Semaphorins: green light for redox signaling? *Sci. STKE* 2002:pe44. 10.1126/stke.2002.155.pe44 12393918

[B43] Wang H. Mu X. Zhou S. Zhang J. Dai J. Tang L. (2014). NEDD9 overexpression is associated with the progression of and an unfavorable prognosis in epithelial ovarian cancer. *Hum. Pathol.* 45 401–408. 10.1016/j.humpath.2013.10.005 24439227

[B44] Xue Y. Bi F. Zhang X. Zhang S. Pan Y. Liu N. (2006). Role of Rac1 and Cdc42 in hypoxia induced p53 and von Hippel-Lindau suppression and HIF1alpha activation. *Int. J. Cancer* 118 2965–2972. 10.1002/ijc.21763 16395716

[B45] Yang Y. Du J. Hu Z. Liu J. Tian Y. Zhu Y. (2011). Activation of Rac1-PI3K/Akt is required for epidermal growth factor-induced PAK1 activation and cell migration in MDA-MB-231 breast cancer cells. *J. Biomed. Res.* 25 237–245. 10.1016/S1674-8301(11)60032-8 23554696 PMC3597073

[B46] Yoon J. Terman J. R. (2018). MICAL redox enzymes and actin remodeling: new links to classical tumorigenic and cancer pathways. *Mol. Cell Oncol.* 5:e1384881. 10.1080/23723556.2017.1384881 29404387 PMC5791864

[B47] Zhang S. S. Wu L. H. Liu Q. Chen K. S. Zhang X. F. (2015). Elevated expression of NEDD9 is associated with metastatic activity in gastric cancer. *Onco Targets Ther.* 8 633–640. 10.2147/OTT.S77904 25792847 PMC4360801

[B48] Zhou S. Xu M. Shen J. Liu X. Chen M. Cai X. (2017). Overexpression of NEDD9 promotes cell invasion and metastasis in hepatocellular carcinoma. *Clin. Res. Hepatol. Gastroenterol.* 41 677–686. 10.1016/j.clinre.2017.04.011 28578938

