# Supplementary figures and images for: NEDD9 Facilitates Hypoxia-Induced Gastric Cancer Cell Migration via MICAL1 Related Rac1 Activation
Source: Front Pharmacol. 2019 Apr 4;10:291. doi: 10.3389/fphar.2019.00291 (PMC6458266; doi:10.3389/fphar.2019.00291)

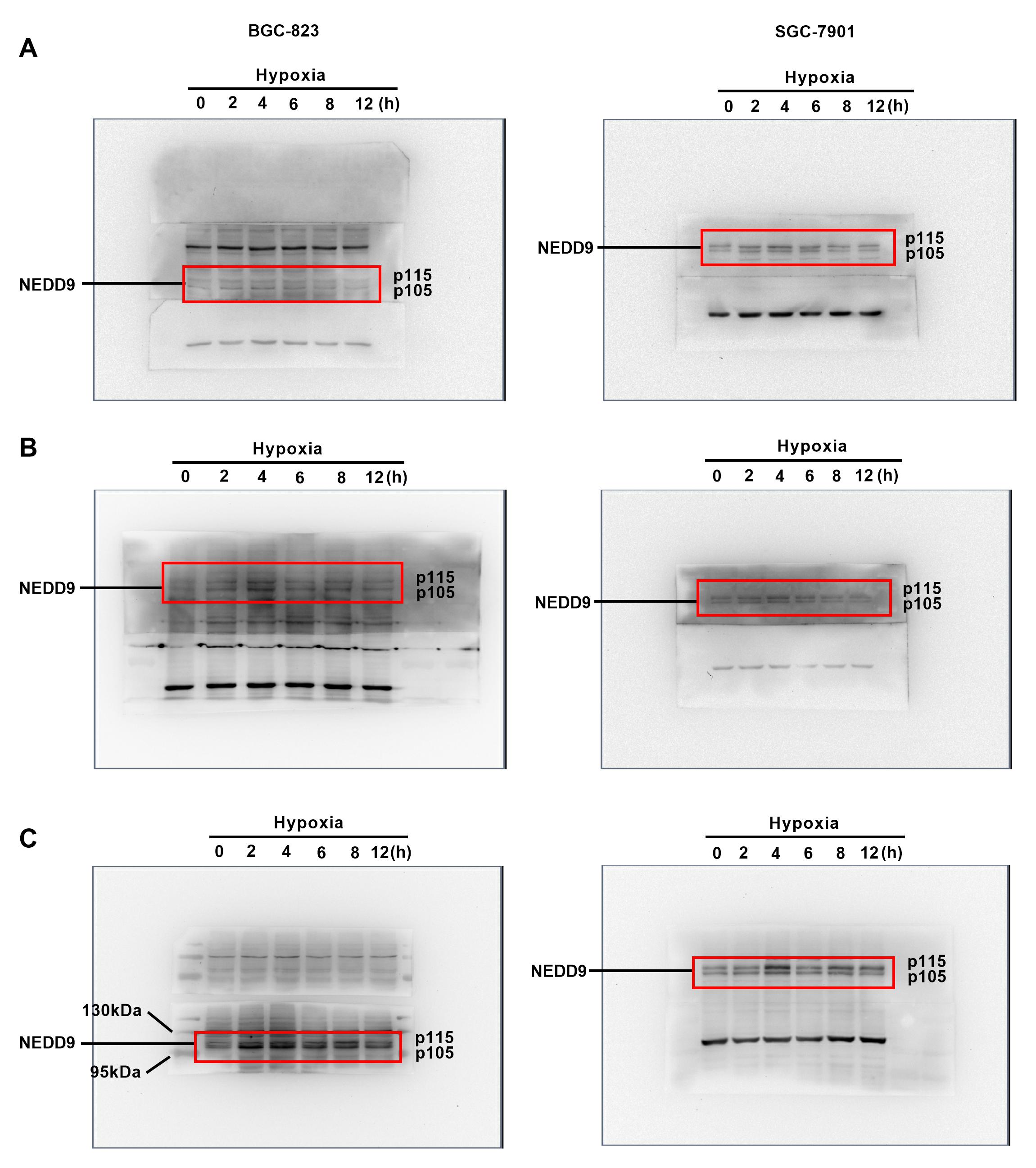

Supplement: FIGURE S1 — Raw data of NEDD9 expression under hypoxia. The membranes were exposed for protein bands. NEDD9 expression was detected three times (A–C). [file Image_1.JPEG]

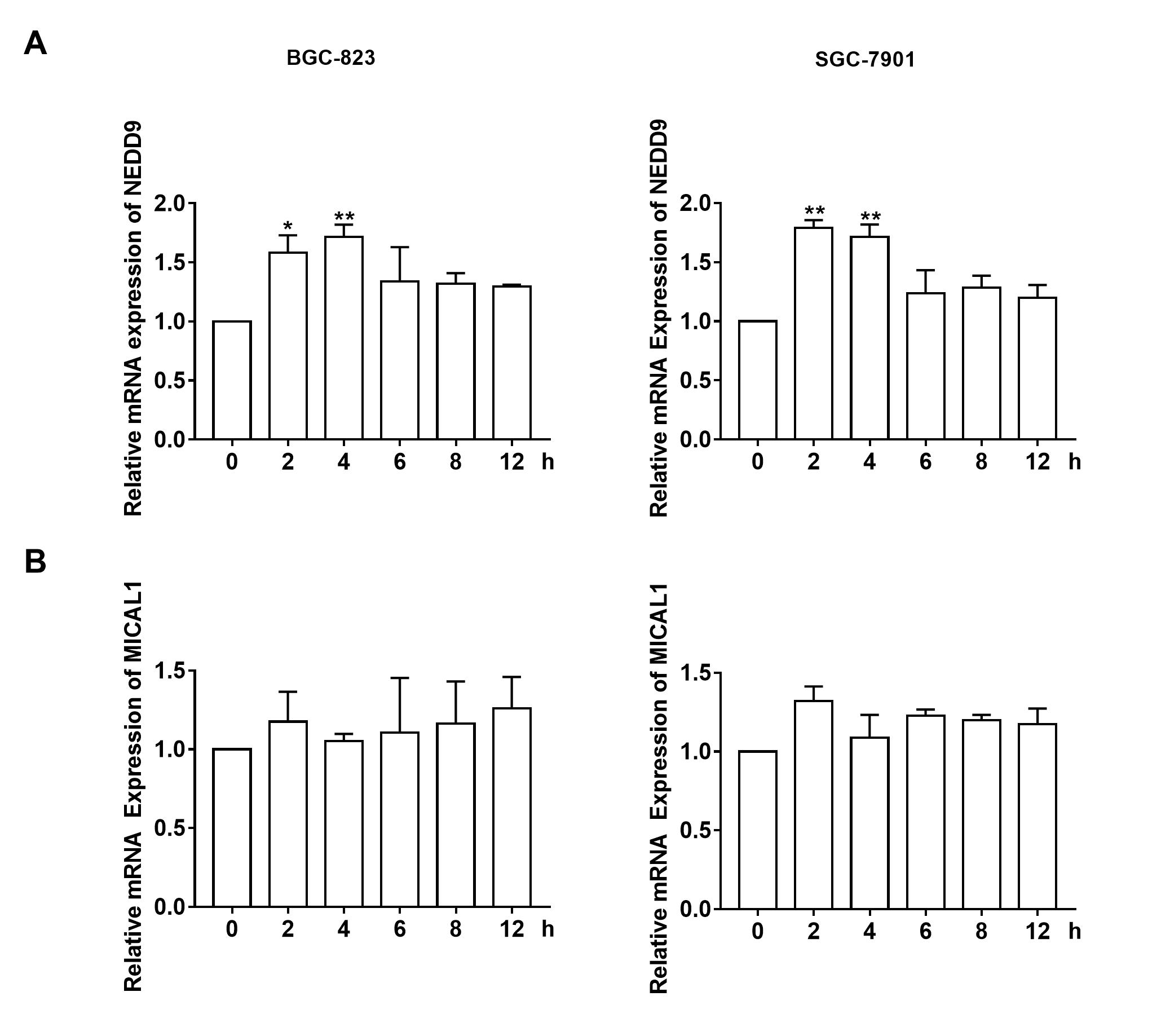

Supplement: FIGURE S2 — Effect of hypoxia on (A) NEDD9 and (B) MICAL1 mRNA expressions. Gastric cancer cells BGC-823 and SGC-7901 were exposed to hypoxia for 12 h and NEDD9 and MICAL1 mRNA levels were determined by qPCR. Afterward, NEDD9 and MICAL1 mRNAs were quantified and normalized against β-actin. ∗P < 0.05, ∗∗P < 0.01, referring to the difference between cells treated with and without hypoxia. [file Image_2.JPEG]

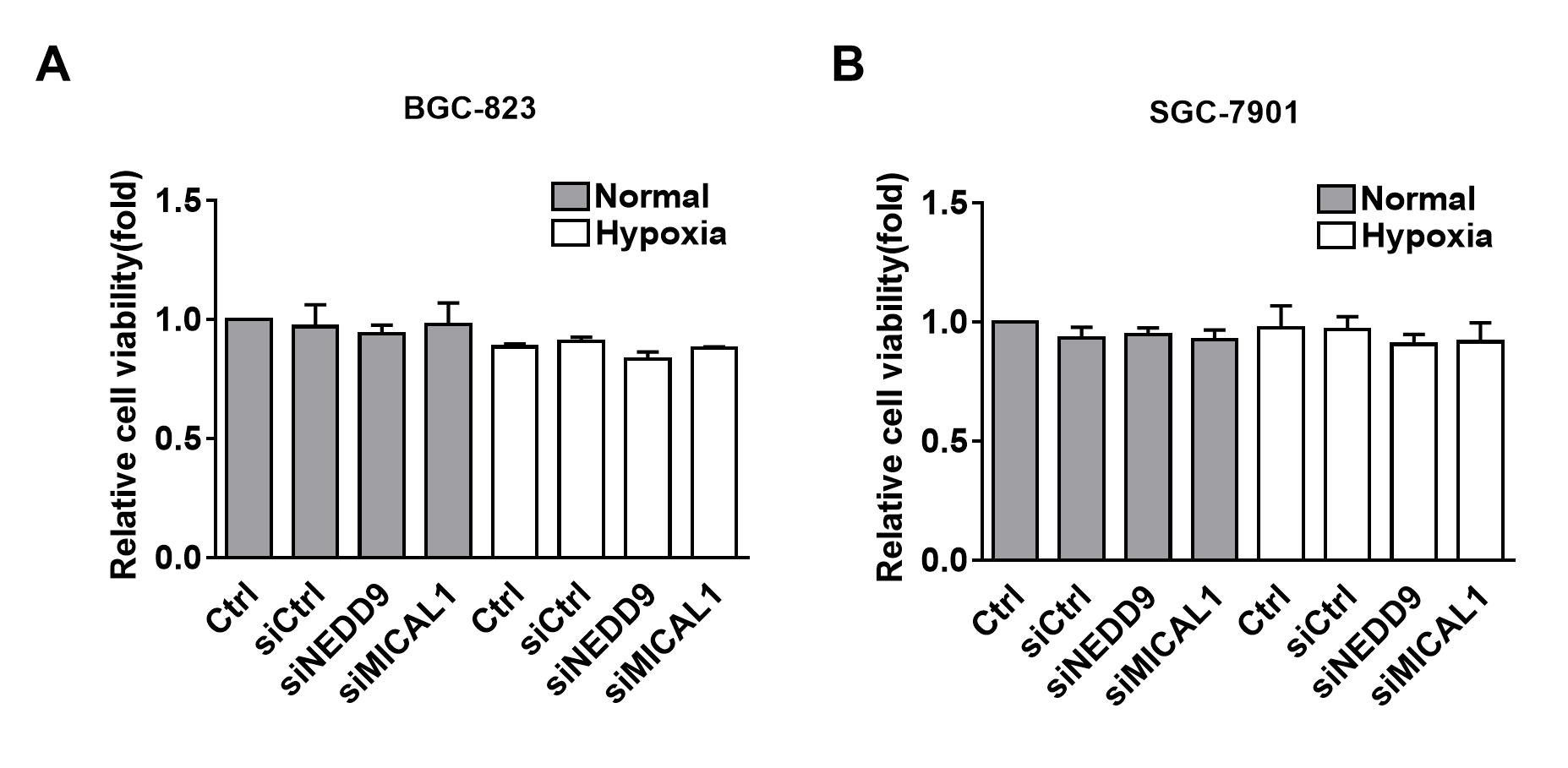

Supplement: FIGURE S3 — Effect of NEDD9 and MICAL1 on cell proliferation under hypoxia. (A) BGC-823 and (B) SGC-7901 cells were transfected with MICAL1 siRNA and NEDD9 siRNA and then they were exposed to hypoxia for 12 h. The cells were incubated with CCK8 and analyzed for cell proliferation. [file Image_3.JPEG]

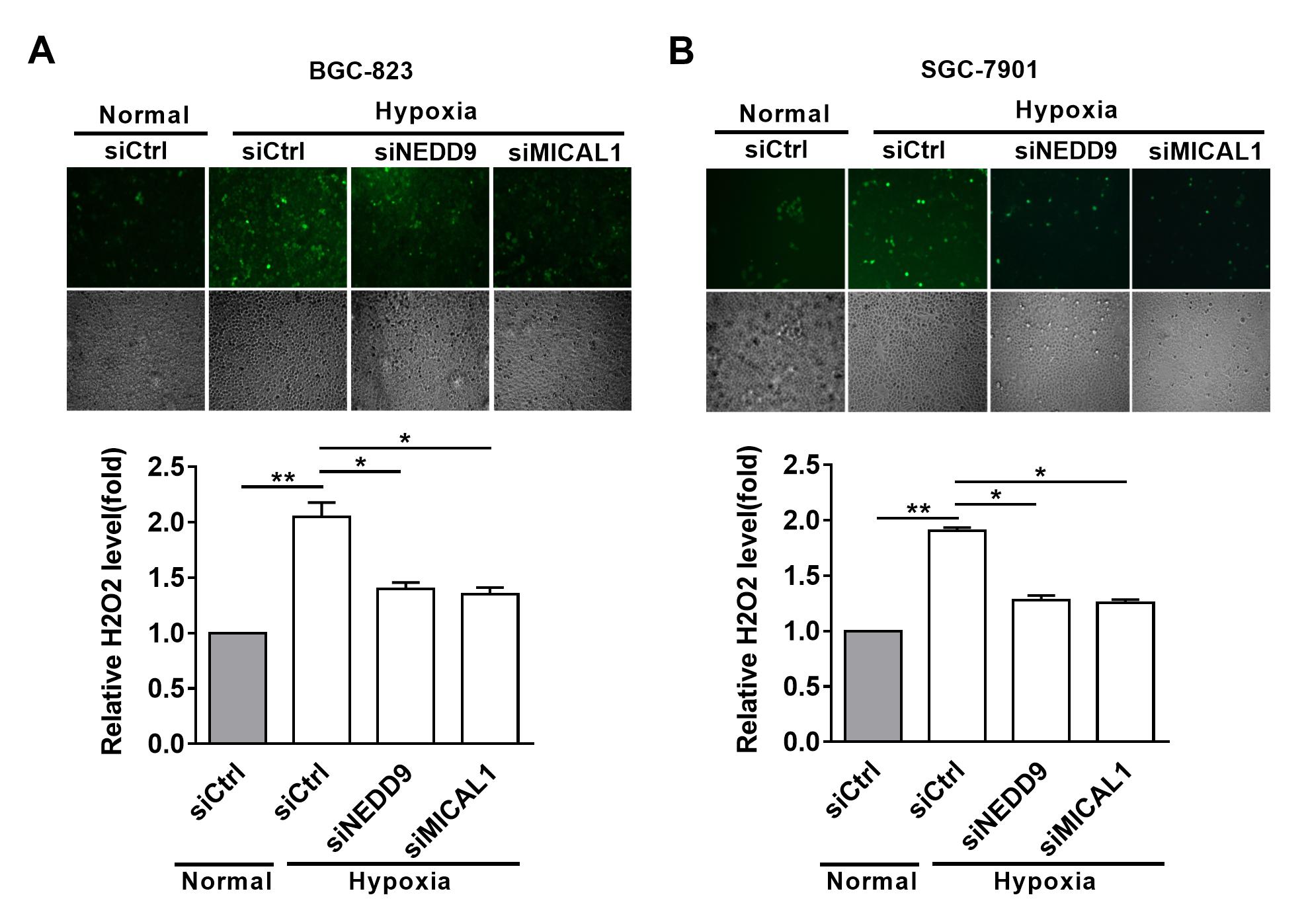

Supplement: FIGURE S4 — Effect of NEDD9 and MICAL1 on ROS production under hypoxia. (A) BGC-823 and (B) SGC-7901 cells were transfected with MICAL1 siRNA or NEDD9 siRNA and then exposed to hypoxia for 4 h. Afterward, the cells were incubated with CM-H2DCFDA and analyzed for ROS generation. ∗P < 0.05, ∗∗P < 0.01. [file Image_4.JPEG]

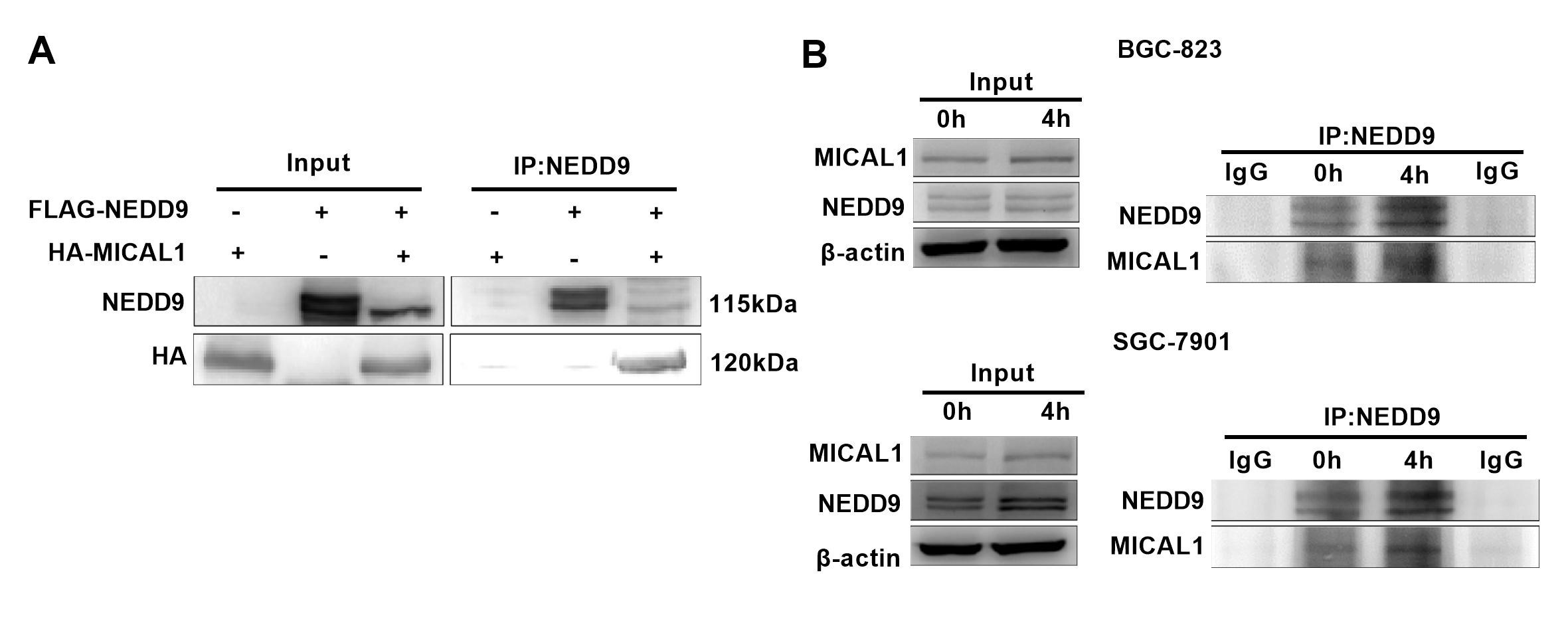

Supplement: FIGURE S5 — NEDD9 interacts with MICAL1 in gastric cancer cells. (A) Immunoprecipitation assay was performed in HEK293T cells that co-transfected with HA-tagged MICAL1 or/and FLAG-tagged NEDD9. (B) Co-immunoprecipitation assay was performed which shows binding of endogenous NEDD9 to MICAL1 in BGC-823 and SGC-7901 cells under hypoxia. [file Image_5.JPEG]

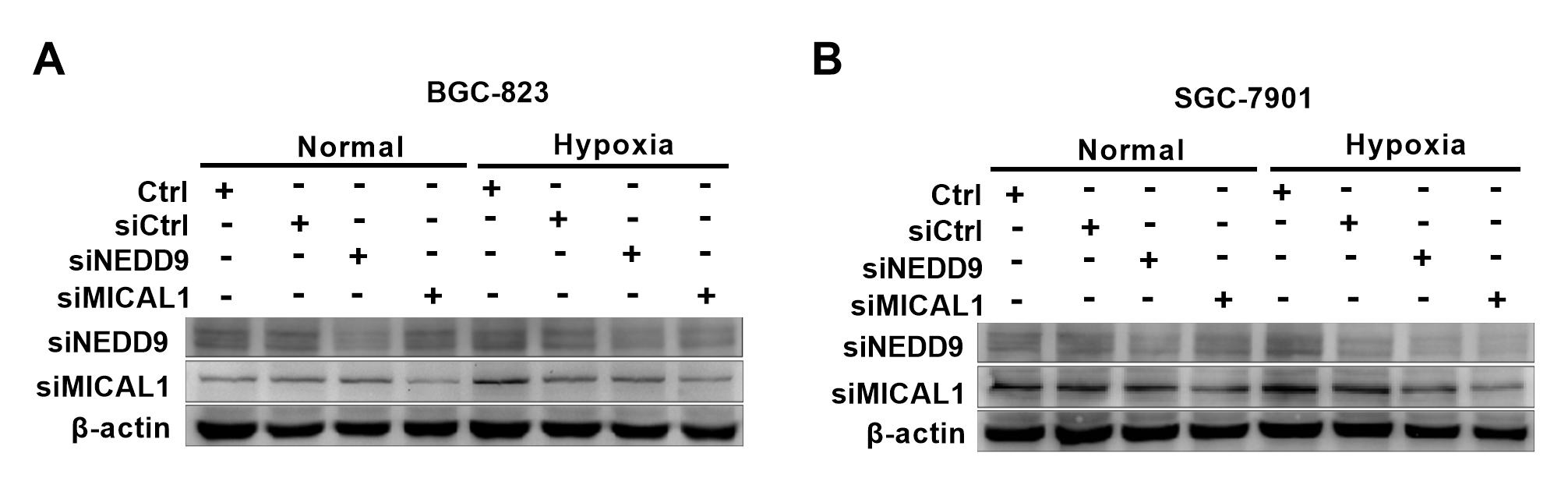

Supplement: FIGURE S6 — Knockout efficiency of NEDD9 and MICAL1. (A) BGC-823 and (B) SGC-7901 cells were transfected with MICAL1 siRNA or NEDD9 siRNA, and then exposed to hypoxia for 4 h. Protein extracted from cells was analyzed by immunoblotting analysis. [file Image_6.JPEG]
